# Different Institutions and Different Values: Exploring First-Generation Student Fit at 2-Year Colleges

**DOI:** 10.3389/fpsyg.2018.00502

**Published:** 2018-04-11

**Authors:** Yoi Tibbetts, Stacy J. Priniski, Cameron A. Hecht, Geoffrey D. Borman, Judith M. Harackiewicz

**Affiliations:** ^1^Curry School of Education, University of Virginia, Charlottesville, VA, United States; ^2^Department of Psychology, University of Wisconsin–Madison, Madison, WI, United States; ^3^Departments of Educational Leadership and Policy Analysis and Sociology, University of Wisconsin–Madison, Madison, WI, United States

**Keywords:** first-generation students, social class, social psychological intervention, values affirmation, cultural mismatch theory

## Abstract

First-generation (FG) college students (students for whom neither parent has a 4-year degree) face a number of challenges as they attempt to obtain a post-secondary degree. They are more likely to come from working-class backgrounds or poverty (Reardon, 2011) and attend lower quality high schools (Warburton et al., 2001) while not benefiting from the guidance of a parent who successfully navigated the path to higher education. FG college students also contend with belonging or “fitting in” concerns due a perceived mismatch between their own values and the values implicit in institutions of higher education (Stephens et al., 2012a). Specifically, prior research has demonstrated that FG college students face an unseen disadvantage that can be attributed to the fact that middle-class norms of independence reflected in American institutions of higher education can be experienced as threatening by many FG students who have been socialized with more interdependent values commonly espoused in working-class populations. The present research examines this theory (cultural mismatch theory) in the understudied context of 2-year colleges and tests if a values-affirmation intervention (i.e., an intervention that has shown promise in addressing identity threats and belonging concerns) can be effective for FG college students at these 2-year campuses. By considering the tenets of cultural mismatch theory in the creation of the values-affirmation interventions we were able to vary different aspects of the intervention in order to examine how its effectiveness may depend on the nature and magnitude of a perceived cultural mismatch. Results from surveying faculty and students at 2-year colleges indicated that compared to traditional 4-year institutions, the norms of 2-year colleges and the motivations of FG students may be different. That is, FG student motives may be more consistent (and thus less mismatched) with the cultural context of 2-year colleges which could result in fewer belonging concerns when compared to FG students at 4-year institutions. This may carry implications for the efficacy of values-affirmation interventions and could help explicate why FG students in the current sample perceived a greater match with their college when they reflected on their interdependent values.

## Introduction

First-generation (FG) college students (students for whom neither parent has a 4-year degree) face a number of challenges as they attempt to obtain a post-secondary degree. They are more likely to come from working-class backgrounds or poverty (Saenz et al., 2007; Reardon, 2011) and attend lower quality high schools (Terenzini et al., 1996; Warburton et al., 2001) while not benefiting from the guidance of a parent who successfully navigated the path to higher education. Compared with continuing generation (CG) college students (students for whom at least one parent has a 4-year degree), FG students struggle in college as they generally have higher drop-out rates, lower grades, and report more difficulty adapting to college (Terenzini et al., 1996; Pascarella et al., 2004; Sirin, 2005). Because parental education is often considered a proxy for social class, the performance discrepancy between FG and CG students is often referred to as the social-class achievement gap (e.g., Jackman and Jackman, 1983; Stephens et al., 2012a; Harackiewicz et al., 2014). In addition to the obvious social and economic barriers that FG students face, they also contend with psychological challenges related to worrying about “fitting in” at college that can impair academic performance (Croizet and Claire, 1998; Ostrove and Long, 2007; Johnson et al., 2011; Smeding et al., 2013; Harackiewicz et al., 2014). This concern about fitting in or belonging in college may be a result of FG students experiencing an unintended identity threat (Stephens et al., 2012a). Stephens et al. (2012a) have demonstrated that FG students face an unseen disadvantage that can be attributed to the fact that middle-class norms of independence reflected in American institutions of higher education can be experienced as threatening by many FG students who have been socialized with more interdependent values commonly espoused in working-class populations. The present research examines this theory (cultural mismatch theory) in the understudied context of 2-year colleges and tests if a values-affirmation (VA) intervention (an intervention that has shown promise in addressing identity threats and belonging concerns) can be effective for FG students at these 2-year campuses.

### Cultural Mismatch Theory

Cultural mismatch theory describes how the academic performance of FG college students may suffer due to a discrepancy between their interdependent values and the independent norms implicit in institutions of higher education (Stephens et al., 2012a). Previous research has shown that whereas university values reflect traditional middle-class norms of independence, FG students tend to endorse interdependent values more prevalent among working-class populations. This carries critical consequences for how FG students experience the culture of higher education. Whereas a culture of independence may be familiar to CG students who have been socialized with more independent norms, it can be experienced as an identity threat for their FG student peers (Stephens et al., 2012b). For example, upon reading a welcoming letter that portrayed their university’s values as more independent (e.g., promoting independent thinking), FG students (but not CG students) showed increased physiological signs of stress (indexed by spikes in cortisol levels) before completing a challenging task (giving a speech; Stephens et al., 2012b). Conversely, when a nearly identical university welcoming letter was framed around promoting interdependent values (e.g., promoting collaboration), FG students showed no signs of increased stress. In addition to feeling more stressed, experiencing the culture of higher education as “mismatched” with one’s own values can impact objective measures of academic performance (Stephens et al., 2012a).

When researchers poll students about their motives for attending college, CG students typically endorse more independent motives and FG students tend to endorse more interdependent motives (e.g., Stephens et al., 2012a; Harackiewicz et al., 2014). Similar to the motives of CG students, administrators from top-tier schools (i.e., colleges/universities ranked among the top 50 by the U.S. News and World Report) tend to emphasize the importance of independent skills (e.g., conducting independent research) over interdependent skills (e.g., conducting collaborative research) as goals for undergraduate education. It is therefore unsurprising that students’ independent motivations (i.e., motives consistent with CG students’ motivations and “matched” with the context of higher education) have positively predicted academic performance in previous research, whereas interdependent motives (i.e., motives consistent with FG students’ motivations and “mismatched” with the context of higher education) have negatively predicted academic performance (Stephens et al., 2012a). In fact, Stephens et al. (2012a) found that the discrepancy between FG and CG students on academic performance was mediated by the varying extents to which they endorsed independent and interdependent motives.

However, the majority of cultural mismatch research has taken place at selective private and flagship public universities, but many FG students attend community colleges and 2-year colleges. Less is known about the nature of cultural mismatch at such institutions, where FG students often comprise a greater proportion of the student population. Thus, one goal of the current project was to examine the extent to which cultural mismatch exists at 2-year institutions that often serve as gateways to 4-year institutions for FG students. By using measures that were originally employed to document cultural mismatch at 4-year institutions, we were able to gain insight into the nature of cultural mismatch in a new context. Furthermore, we implemented a VA intervention in order to address the identity threat and belonging concerns that may result from the cultural mismatch that FG students experience at these 2-year institutions. By considering cultural mismatch in the construction of the VA intervention we were able to vary different aspects of the intervention assignments in order to shed light on how its effectiveness may depend on the nature and magnitude of an experienced mismatch.

### Values Affirmation

The second goal of the current project was to assess the efficacy of different VA interventions for FG students. VA is typically implemented as a writing intervention that instructs students to reflect on their important values in evaluative and potentially threatening contexts (Cohen and Sherman, 2014). For students facing belonging concerns and potential identity threats (e.g., FG students), affirming personal values may be a way of reestablishing a feeling of self-integrity and self-worth thereby bolstering them from the negative effects of feeling like they do not belong. VA interventions have proven effective at improving the academic performance of students who feel like they don’t belong (Layous et al., 2017) including FG students and other traditionally underrepresented students (Cohen et al., 2006; Sherman et al., 2013; Hanselman et al., 2014; Harackiewicz et al., 2014; Tibbetts et al., 2016a).

Recent research at a flagship 4-year university noted that FG students who wrote about the personal importance of values related to independence and academics (e.g., *learning and gaining knowledge, curiosity, independence*) performed better in a biology course (Study 1) and on a standardized math test (Study 2; Tibbetts et al., 2016a). Furthermore, FG students who wrote about their independence were less concerned about their academic background at the end of the semester (Study 1). It may be that writing about independence in a flagship university characterized by independent norms enabled FG students to feel more comfortable about their background, leading to improved academic performance. Conversely, VA studies have also noted that writing about interdependence can be beneficial for some groups of students. For example, Covarrubias et al. (2016) noted that an interdependent-focused VA intervention improved the academic performance of Latino college students. Furthermore, Tibbetts et al. (2016a) noted that although writing about independence mediated VA effects for FG students, these students often wrote about independence in *addition* to writing about their interdependence. Thus, in the present study we included VA assignments that emphasized independence, interdependence, or both independence *and* interdependence. It may be that the effectiveness of different VA interventions (e.g., independent or interdependent VA interventions) is predicated on which norms are more salient at the academic institution. If 2-year college norms are similar to 4-year universities in that they emphasize independence over interdependence then the independent VA condition may be more effective for FG students in the current study. However, if the norms of 2-year colleges are more interdependent than independent (contrary to what is typically found at 4-year institutions but consistent with typical FG student values), it may be that the interdependent VA condition is more effective for FG students. Including a condition that emphasized both independence and interdependence allowed us to test if it is critical to affirm both kinds of values within a VA intervention. If 2-year colleges are perceived to equally espouse independent and interdependent values, it could be maximally beneficial for students to reflect on both independence and interdependence.

Additionally, given that FG students are often socialized with working-class norms of interdependence and then required to attend institutes of higher education that emphasize traditional middle-class norms of independence, it may be beneficial for them to reflect on both of their cultural experiences. Research on biculturalism indicates that individuals with two cultural identities can experience psychological benefits (e.g., belonging) from orienting to both cultures (i.e., bicultural integration) rather than a single one (Nguyen and Benet-Martínez, 2013). Furthermore, recent research indicates that individuals with multiple cultural backgrounds (e.g., African–American students) can leverage their diverse cultural experiences to improve academic outcomes (Brannon et al., 2015). For example, Brannon et al. (2015) demonstrated that an independent self-schema is chronically activated within United States educational settings but that incorporating more culturally relevant interdependent practices may benefit bicultural students (e.g., African–American students) who traditionally respond more independently or interdependently depending on the dominant cues in a given setting. Thus, we included conditions that emphasized independence, interdependence, or both and measured institutional norms of independence and interdependence to provide us more context to interpret treatment effects.

We hypothesized that, consistent with prior research (e.g., Tibbetts et al., 2016a), FG students would perform better in conditions that emphasized independent values (e.g., the independent VA condition and combined VA condition). However, if the norms institutional norms implicit at the 2-year colleges differed from traditional 4-year universities (i.e., if norms 2-year university norms are more interdependent), we predicted that VA conditions that emphasized interdependence (e.g., the interdependent VA condition and combined VA condition) may be most effective for FG students.

### Setting

This field study was conducted in the context of 2-year colleges in a Midwestern state. This state’s public university system is comprised of both 4-year and 2-year campuses, with the 2-year campuses providing a primary pipeline for transfer to the 4-year campuses, especially for students who are not academically prepared or financially able to begin higher education at a 4-year institution. These institutions often serve more FG students than traditional 4-year colleges and universities. Whereas FG students are typically underrepresented at 4-year institutions, they comprise the majority of students (roughly 71%) at public 2-year institutions (Cataldi et al., 2018). However, even at these 2-year institutions, FG students graduate at a significantly lower rate than their CG student peers (Cataldi et al., 2018).

By charging the lowest tuition rates in the State College System and admitting every qualified student who applies (i.e., high school graduates or GED holders with a minimum of 17 college preparatory credits and a registered ACT or SAT score), the 13 two-year campuses (also called freshman/sophomore campuses) provide a more affordable and accessible alternative to enrolling in a 4-year baccalaureate program. Furthermore, in order to encourage students to eventually obtain a bachelor’s degree, the state’s university system has implemented a “Guaranteed Transfer Program.” Students who complete the required number of credits to earn an associate’s degree with a minimum grade point average of 2.0 are guaranteed admission as a junior to one of the state’s 4-year colleges. For many students, starting at these 2-year colleges is critical to eventually obtaining a bachelor’s degree. Thus, the 2-year college system serves many of the roles of community colleges in other states, but with a more concrete pathway to a 4-year degree.

However, similar to traditional community colleges, many students struggle in this 2-year college system, in part, because they face additional challenges less commonly encountered by students at 4-year institutions. For example, in the current sample nearly four in five students (79%) work during the school year with students reporting an average of 17 h of work per week. Additionally, over half of the current sample is from traditionally underrepresented populations [e.g., FG or underrepresented ethnic minority (URM) students] in higher education. This pattern is consistent with research noting that traditional community college students are more likely to come from disadvantaged backgrounds, have families to support, and/or work full-time (Horn and Nevill, 2006; Goldrick-Rab, 2010). These challenges may contribute to higher dropout rates and delayed degree completion for many of these students (Bailey T. et al., 2006; Bailey T.R. et al., 2006).

For these reasons, we attempted to address the social-class achievement gap in 2-year colleges by implementing a VA intervention in a double-blind, randomized field experiment. The experiment was conducted in introductory biology and psychology courses (five biology, 10 psychology) taught by 11 instructors across six campuses. In addition to addressing the social-class achievement gap, working with these schools provided us the opportunity to examine the cultural mismatch theory in a novel context. The tenets of cultural mismatch theory have been founded upon research conducted at mostly selective private and flagship public universities. Examining the nature of cultural match and mismatch at 2-year colleges, where there might be a different institutional culture, could provide valuable insight into how the college experience of FG students (and their experience of institutional culture) varies by type of institution.

## Study 1A: Assessing Cultural Mismatch at the 2-Year Colleges

To assess cultural mismatch at the 2-year colleges we employed the same measures that were used to document cultural mismatch in previous research at 4-year institutions (e.g., Stephens et al., 2012a). In accordance with prior cultural mismatch research, we measured institutional norms at the 2-year colleges (with surveys asking course instructors about their colleges’ values and norms) and students’ motivations for attending college to examine if discrepancies between perceived norms and student motivation existed. Furthermore, given that previous research has noted that a perceived mismatch could contribute to a lack of perceived belonging for FG students, relative to their CG peers in 4-year contexts (e.g., Harackiewicz et al., 2014), we included several measures related to belonging and perceived match with the college.

### Faculty Sample – Measuring University Norms

Given that most of the research on cultural mismatch between personal values and university norms has been conducted at selective 4-year institutions we felt that it was important to measure institutional norms in the present context of the 2-year colleges. Indeed, conversations with the provost of the 2-year colleges system during the planning stages of the study suggested that the 2-year colleges may value interdependence more than typical 4-year universities. The provost noted that small class sizes that encourage student-faculty interactions and a commitment to creating a school community spirit are essential to building the inclusive environment that the 2-year colleges strive to provide.

#### Participants

In order to assess whether and how the school culture at the 2-year colleges differed from that of 4-year institutions, we measured the extent to which the 2-year colleges were characterized by norms of independence or interdependence with a survey that was e-mailed directly to a small set of course instructors who have worked with our research group over the last three semesters. Of the 26 course instructors we contacted, 18 responded (nine males and nine females across three different departments: nine biology faculty, five psychology faculty, and four chemistry faculty). Instructor responses were then compared to the responses from administrators from 4-year universities documented in the Stephens et al. (2012a) study that first documented a cultural mismatch for FG students.

#### Methods

The extent to which the culture of the 2-year colleges was characterized by norms of independence or interdependence was assessed with two measures used by Stephens et al. (2012a). The first measure presented a list of 12 institutional expectations, half reflecting norms of independence (*Learn to express oneself, Learn to be a leader, Learn to influence others, Learn to do independent research, Learn to work independently, Learn to solve problems on one’s own*) and half reflecting norms of interdependence (*Learn to work together with others, Learn to do collaborative research, Learn to listen to others, Learn to be a team player, Learn to adjust to others’ expectations, Learn to ask others for help*) and asked instructors to pick the five most important expectations (out of the 12 on the list).

The second task presented six pairs of institutional expectations, with each pair divided into one statement reflecting an independent norm and one statement reflecting an interdependent norm (*Being independently motivated* vs. *Being motivated by others’ high expectations, Working independently* vs. *Working collaboratively in groups, Conducting independent research projects* vs. *Conducting collaborative research projects, Paving their own innovative pathways* vs. *Following in the footsteps of accomplished others, Challenging the norms or rules* vs. *Considering the norms or rules, Developing personal opinions* vs. *Appreciating the opinions of others*). For each pair, instructors were asked to choose the one statement that best reflected the dominant cultural norm at their university.

#### Results

For the first task, in which instructors picked the five most important expectations that characterized their university’s culture, we examined the percentage of times that each item was selected by instructors, separately by independent and interdependent items, and averaged across the six items. Independent items (e.g., Learn to be a leader) were selected on average, 39% of the time. Interdependent items (e.g., Learn to work together with others) were selected as one of the five most important expectations, on average, 45% of the time. This pattern is opposite to the pattern observed by Stephens et al. (2012a), where independent items were selected more often than interdependent items at selective colleges and universities (53% for independent items, 30% for interdependent items; see **Table 1**).

**Table 1:** Study 1A: Percent of independent and interdependent items endorsed by 2-year colleges’ instructors and administrators in Stephens et al. (2012a) study.

Survey items	% Items selected (Study 1A)	% Items selected (Stephens et al., 2012a)
**Independent**		
Learn to express oneself	72%	74%
Learn to work independently	61%	46%
Learn to solve problems on one’s own	61%	60%
Learn to do independent research	28%	55%
Learn to influence others	6%	17%
Learn to be a leader	6%	68%
Mean percent	39%	53%
**Interdependent**		
Learn to work together with others	100%	58%
Learn to listen to others	50%	36%
Learn to do collaborative research	39%	46%
Learn to ask others for help	39%	12%
Learn to be a team player	28%	25%
Learn to adjust to others’ expectations	11%	2%
Mean percent	45%	30%


For the second task, in which instructors chose between two statements, we examined the percentage of independent choices made by instructors. Independent items (e.g., Working independently) were selected, on average, 49% of the time, meaning that interdependent items (e.g., Working collaboratively in groups) were selected, on average, 51% of the time. This pattern (i.e., roughly equivalent endorsement of interdependent and independent items) is inconsistent with the pattern noted by Stephens et al. (2012a) in which independent items were much more strongly endorsed at selective colleges and universities (70% for independent items; see **Table 2**).

**Table 2:** Study 1A: Percent of independent expectations selected by 2-year colleges’ instructors and university administrators in Stephens et al. (2012a) study.

Pairs of survey items	% Independent items	
	
	Study 1A	Stephens et al., 2012a
Being independently motivated	83%	92%
Being motivated by others’ high expectations		
Paving their own innovative pathways	72%	86%
Following in the footsteps of accomplished others		
Developing personal opinions	61%	60%
Appreciating the opinions of others		
Working independently	33%	55%
Working collaboratively in groups		
Conducting independent research projects	28%	71%
Conducting collaborative research projects		
Challenging the norms or rules	17%	53%
Considering the norms or rules		
Mean percent	49%	70%


### Student Sample – Measuring Student Motives

Students’ motives to attend college were measured as part of the current VA study. Using methods previously documented in cultural mismatch research (e.g., Stephens et al., 2012a; Harackiewicz et al., 2014), we surveyed students about their independent and interdependent motives for attending college. Additionally, because previous research has noted that a perceived mismatch between FG students’ motivation and their college context could result in a decreased sense of belonging we included measures of college belonging, academic and social concern (Sherman et al., 2009), and perceived match.

#### Participants

In order to be included in the study, students had to provide consent for access to their academic records, complete at least one of the VA writing assignments, and complete the course. Of the 518 students enrolled in the participating introductory biology and psychology courses, 438 were retained in the final sample. Twenty-six students did not give consent for access to academic records, seven did not complete either VA assignment, and 47 students dropped the course [consent rate, VA assignment completion, and dropout rate did not vary significantly by underrepresented minority (URM) status, FG status, or experimental condition].

Participants were 62% female (38% male), 57% FG students (43% CG students), and 57% psychology students (43% biology students) with an average age of 19.97 (*SD* = 3.67). The sample self-identified as 84% White, 7% Hispanic, 4% Asian/Asian American, 2% African American, 2% Southeast Asian, and 1% Native American. In this study URM students were defined as being Hispanic, African American, Southeast Asian, or Native American. Of the 251 FG students 16% (*N* = 41) were also URM students and of the 187 CG students 6% (*N* = 12) were URM students, indicating that URM students were somewhat overrepresented in the FG group, as expected, χ^2^(1, *N* = 438) = 9.91, *p* = 0.002. Additionally, these participants constitute the sample used in Study 1B.

#### Methods

Consistent with prior research, student motives, perceived match, and belonging were assessed at the beginning of the semester (the 1st week of class), prior to any intervention implementation (Harackiewicz et al., 2014).

##### Motives

Independent motives (e.g., “Become an independent thinker,” α = 0.79) and interdependent motives (e.g., “Give back to my community,” α = 0.76) were assessed on a 1 (Not at all important) to 7 (Very important) Likert scale in response to the prompt “*I am motivated to attend college because I want to…*” (see **Table 3** for full scale). This is consistent with more recent studies (e.g., Stephens et al., 2014) but different than previous research that assessed these motives by asking students to select from a checklist which of the motives represented “*a very important reason for completing your college degree*” (Stephens et al., 2012a; Harackiewicz et al., 2014).

**Table 3:** Study 1A: Percent of interdependent and independent items endorsed by first-generation, continuing-generation, majority first-generation, and minority-first-generation students.

Survey items	All CG students	All FG students	Majority-FG students	Minority-FG students
**Interdependent items**				
Help my family out after I’m done with college	4.69 (1.73)	5.29 (1.65)	5.09 (1.62)	6.34 (1.39)
Be a role model for people in my community	5.50 (1.38)	5.71 (1.50)	5.59 (1.51)	6.35 (1.28)
Show that people with my background can do well	4.97 (1.77)	5.63 (1.53)	5.45 (1.58)	6.53 (0.84)
Give back to my community	5.10 (1.49)	5.40 (1.57)	5.24 (1.58)	6.24 (1.22)
Provide a better life for my own children	5.85 (1.58)	6.25 (1.34)	6.14 (1.43)	6.82 (0.54)
Scale mean	5.22 (1.08)	5.66 (1.11)	5.50 (1.10)	6.46 (0.75)
**Independent items**				
Become an independent thinker	5.89 (1.09)	5.89 (1.37)	5.77 (1.40)	6.51 (0.98)
Explore new interests	5.41 (1.49)	5.61 (1.39)	5.49 (1.41)	6.22 (1.09)
Learn more about my interests	6.08 (1.05)	6.21 (1.08)	6.14 (1.12)	6.54 (0.75)
Expand my understanding of the world	5.90 (1.18)	6.01 (1.18)	5.90 (1.21)	6.56 (0.78)
Scale mean	5.82 (0.92)	5.93 (1.00)	5.83 (1.02)	6.46 (0.67)


**CG, continuing-generation; FG, first-generation*.*

##### Academic and social concerns

As in the Harackiewicz et al. (2014) study we also included a measure of academic and social concerns (Sherman et al., 2009) that consisted of the following four items: “In college, I sometimes worry that people will dislike me,” “In college, I worry that people will think I’m unintelligent if I do poorly,” “I am usually confident that others will have a good impression of my ability,” “In college, I often get nervous and worried when I talk to people” (α = 0.74).

##### Perceived match

Perceived match with the university was measured with two items (“I feel that the values of 2-year colleges system X^1^ align with those that I was raised with,” and “I feel that my values and goals are well matched with those of 2-year colleges system X,” α = 0.82).

##### College belonging

College belonging was measured with two items (“I belong in college” and “I feel like college is a good fit for me,” α = 0.83).

#### Results

We tested whether students’ motives, academic and social concerns, perceived match, or college belonging varied at baseline as a function of students’ generational status and/or URM status with two orthogonal *Demographic* contrasts. The first contrast, labeled the *Generational Status* contrast, tests differences between CG students (-2) and all FG students, both majority (+1) and minority (+1). The second contrast, labeled the *URM Status* contrast, tests differences between Majority-FG students (+1) and Minority-FG students (-1) with CG students being set to 0. This second contrast was included to test for differences among FG students based on their ethnic minority status. There were not enough Minority-CG students (*N* = 12) to test a 2 (CG vs. FG) × 2 (Majority students vs. Minority students) intersectional model as in Harackiewicz et al. (2016); therefore these Minority-CG students were always included in the CG group^2^.

The analyses on students’ motives revealed that FG students more strongly endorsed both independent, *t*(435) = 2.99, *p* = 0.003, β = 0.16, and interdependent motives, *t*(435) = 6.33, *p* < 0.001, β = 0.34, compared to CG students. Furthermore, Minority-FG students tended to endorse more independent motives, *t*(435) = 3.87, *p* < 0.001, β = -0.21, and interdependent motives, *t*(435) = 5.26, *p* < 0.001, β = -0.28, compared to Majority-FG students, see **Table 3**. The results of the *Generational Status* contrast are somewhat surprising in that the pattern on independent motives is in the opposite direction of previous research (e.g., Stephens et al., 2012a; Harackiewicz et al., 2014). That is, whereas previous research has found that CG students more strongly endorse independent motives for attending college, in the present sample it was FG students who more strongly endorsed both independent and interdependent motives for attending college.

First-generation students did not report significantly different levels of academic and social concerns, perceived match, or college belonging when compared to CG students, *p* > 0.08. A significant effect of the *URM Status* contrast indicated that Majority-FG students reported significantly higher levels of academic and social concerns, *t*(435) = 3.12, *p* = 0.002, β = 0.17, when compared to Minority-FG students (see **Table 4** for means of all baseline variables). No other effects were significant.

**Table 4:** Study 1B: Means and standard deviations of basline psychological, demographic, and descriptive measures.

Measure	All CG students	All FG students	Majority-FG students	Minority-FG students
Academic and social concerns	3.58 (1.31)	3.56 (1.41)	3.68 (1.37)	2.95 (1.49)
Independent motives	5.82 (0.92)	5.93 (1.00)	5.83 (1.02)	6.46 (0.67)
Interdependent motives	5.22 (1.08)	5.66 (1.11)	5.50 (1.10)	6.46 (0.75)
Perceived match	5.01 (1.19)	5.15 (1.60)	5.12 (1.17)	5.29 (1.09)
College belonging	5.97 (1.17)	5.99 (1.11)	5.94 (1.15)	6.22 (0.87)
ACT score	21.56 (3.79)	20.04 (3.41)	20.34 (3.30)	18.48 (3.57)
Number of AP/IB courses taken	1 (1.63)	0.65 (1.12)	0.69 (1.15)	0.44 (0.92)
Age	19.63 (3.30)	20.23 (3.92)	20.08 (20.08)	20.95 (20.95)
Income	5.09 (1.91)	4.01 (1.99)	4.09 (1.96)	3.57 (2.12)
	(50–75K)	(35–50K)	(35–50K)	(35–50K)
% Free and reduced lunch	28% (12.93%)	31% (12.77)	31% (12.50%)	36% (13.77%)
% Employed during the semester	86%	74%	76%	68%
Hours worked per week	18.14 (11.55)	16.52 (16.52)	16.56 (13.19)	16.28 (14.25)


**CG, continuing-generation; FG, first-generation.**

### Discussion of Study 1A

Taken together, these results suggest that compared to typical 4-year universities, the norms of 2-year colleges and the motivations of FG students are different, and thus there may not be as great a mismatch for FG students at these schools.

For example, the results of the faculty survey suggest that contrary to previous research in 4-year contexts, 2-year colleges may be more accurately characterized as emphasizing norms of interdependence at least as much as they emphasize independence. Of course, one limitation of the present set of faculty survey results is that they are based on a very small sample (18). We would like to measure these norms at other 2-year or community colleges before generalizing this particular set of results. However, we believe these data at least suggest that some institutions of higher education value norms of interdependence to a similar (or great) extent as independence. This carries important consequences for cultural mismatch theory. Stephens et al. (2012a) posit that the underperformance of FG students is due, in part, to their goals being inconsistent with university norms.

The analysis of the student survey further bolstered the notion that cultural mismatch may be different at 2-year institutions when compared to 4-year colleges. FG students at the 2-year colleges did not show the same psychological and motivational patterns as the FG students in previous research documenting cultural mismatch. For example, contrary to prior research (e.g., Stephens et al., 2012a; Harackiewicz et al., 2014), FG students in the current sample did not report lower endorsement of independent motives or greater academic and social concerns when compared to CG students. Similarly, FG students did not perceive less match or report less college belonging when compared to their CG peers suggesting that the general belonging concerns that FG students face at 4-year universities may not afflict FG students in this 2-year college context.

It is also noteworthy that Minority-FG students reported even greater endorsement of both independent and interdependent motives along with lower levels of academic and social concerns, when compared to Majority-CG students. These results indicate the importance of an intersectional approach when assessing motivational profiles (Harackiewicz et al., 2016), in addition to carefully considering how the student experience varies across different kinds of academic institutions.

More generally, the absence of a mismatch between FG student motivation and the cultural norms of the 2-year colleges may be one reason why FG students did not report less college belonging or more academic and social concerns at the beginning of the semester in comparison to their CG student peers. Whereas FG students at 4-year institutions confront a cultural mismatch that is experienced as stressful (Stephens et al., 2012a) resulting in belonging concerns, FG students at the 2-year colleges may not experience the negative effects associated with cultural mismatch. This may impact the effectiveness of the various VA interventions implemented as part of Study 1B.

## Study 1B: Implementing a Values-Affirmation Intervention at the 2-Year Colleges

In an attempt to address the social-class achievement gap at the 2-year colleges we implemented various VA interventions to the same sample described in Study 1A.

### Overview of Procedure

As noted in student survey from Study 1A, we collected baseline measures of students’ motives for attending college, college belonging, academic and social concerns, and perceived match during the 1st week of the course. Concurrently, we also collected basic demographic information and student consent.

Students were blocked on URM status, FG status, gender, and course (biology or psychology) and were randomly assigned to one of four conditions in a double-blind design. We implemented the VA intervention twice over the course of the semester in the form of an in-class writing assignment the 2nd week of the course (time 1) and as a homework writing assignment at approximately the 8th week of the semester (time 2). Thus, each student had the opportunity to complete either two values-affirmation writing exercises or two control writing exercises of similar format and length. At approximately the 13th week of the semester, we collected the same measures that were collected at baseline with the addition of several new 2-year college specific scales. Final course grades and students’ enrollment status were obtained from students’ academic records. Missing data (less than 10% on each measure) was handled with predictive mean matching multiple imputation (Kaplan and Su, 2016).

#### Values-Affirmation Intervention

The VA intervention was administered as follows: All students were assigned two writing assignments, for credit, over the course of the semester. The first assignment was administered as an in-class writing assignment the 2nd week of the course. Course instructors received the VA assignments from study personnel and distributed personally addressed manila envelopes containing the assignment (which had been assigned in advance, based on the randomized blocked design) to students. As instructors passed out the assignment, a PowerPoint slide with the following text was shown to students:

As you know, classes can be pretty writing-intensive. The purpose of this short exercise is to get you warmed-up and thinking about writing again. For the rest of the semester, you’ll be writing mostly about biology/psychology, but for now you’re just going to write about yourself. Even though I’m grading this only on completion, please give it your full effort and take this opportunity to get your brain working again. I want you to feel comfortable with this type of personal writing, so I’ve asked an independent group to check for completion of this writing exercise so that it can be confidential. They will let me know if you have thoughtfully completed the exercise so that you can receive course credit for it. I will never see it.

These instructions ensured that students knew the assignment came from their instructors and was required for course credit, but that the content of their work would not be evaluated by their instructors and would remain confidential. These conditions satisfy the implementation criteria for values affirmation (Cohen et al., 2006). Similar details were included on the cover page of each writing assignment.

Although there were multiple versions of the writing assignments, the envelopes and formatting of the assignments were similar. Students in each condition received a four-page packet that included the same cover page. The second page of each packet listed values that students were instructed to circle. Students in the affirmation conditions were instructed to circle the two or three of the values that were most important to them and write why they were important to them on the third page of the packet. Students in the control condition were instructed to circle the two or three values that were least important to them and write why they might be important to someone else on the third page of the packet. The directions also instructed students to focus on their thoughts and feelings and to not worry about spelling or grammar. In order to encourage further reflection on their values, the fourth and final page of the packet asked students to look at the values they had previously selected and either list the top two reasons why these values were important to them (VA condition) or the top two reasons why someone else might pick these values as important (control condition). Finally, the fourth page ended by asking students to indicate their agreement with several items intended to prompt further reflection about the values (e.g., *In general, I try to live up to these values* in the affirmation conditions vs. *In general, some people try to live up to these values* in the control condition). After completing the writing, students put the assignments back in the manila envelope, ensuring that course instructors and study personnel remained blind to condition when collecting and handling the data. Once their class concluded, instructors returned the writing assignments to study personnel in a remote area of the school (the mailroom) where students were unlikely to see the exchange. If students were not present during this class time they were given the opportunity to complete the assignment online via a course management website (Desire2Learn). This procedure was similar to those developed and validated in past research (Cohen et al., 2006; Miyake et al., 2010; Harackiewicz et al., 2014).

The second writing exercise was delivered as a homework assignment via the course management site that was customized so students could receive private, individualized links to writing assignments (corresponding to their condition). The links students clicked on opened to a Qualtrics page where they could complete the same writing assignment administered at time 1 with the exception of two new “neutral” values that were utilized at time 2 (see “Experimental Conditions” section for details). After each VA administration study personnel gave instructors a list of which students completed the writing assignments. This process ensured that instructors could assign course credit but remain blind to experimental condition.

Consistent with prior VA research (Tibbetts et al., 2016a) all essays were coded with a binary independent and interdependent coding scheme (i.e., hand-coded by multiple researchers) to detect the presence or absence of independent and interdependent written themes. Essays were identified as including themes of independence if the writing including (a) valuing an activity because it is done alone, (b) explicitly expressing the value of independence for the self, or (c) any related thoughts showing that the participant values his or her own autonomy (i.e., the ability to make her or his own decisions and have her or his own ideas and opinions). Essays were identified as including themes of interdependence if the writing included (a) valuing an activity because it is done with others, (b) feeling part of a group of people because of a certain value or while engaging in a certain activity, or (c) any related thoughts on the subject of one’s interdependence, such as being affiliated with or liked by others.

#### Experimental Conditions

Participants were randomly assigned to receive one of four writing packets, which differed by values list and writing instructions, depending on the condition. For the first VA administration (time 1), participants in the *Control* condition received selected 2–3 of their least important values from a list of 12 values and wrote about why the circled values might be important to someone else (consistent with previously documented VA control conditions; e.g., Harackiewicz et al., 2014).

The three treatment conditions (*Independent VA, Interdependent VA*, and *Combined VA*) were designed to elicit varying amounts of independent or interdependent writing. Previous research has noted that some selected values (e.g., *independence, curiosity*) correspond with more independent writing and that other values (e.g., *relationships with friends and family, belonging to a social*) correspond with more interdependent writing (Tibbetts et al., 2016a). By restricting the number of values that participants can select to independent or interdependent values (along with two rarely selected “neutral values”; *being good at art*, and *government and politics*) researchers have demonstrated the capacity to influence the extent to which participants write about independence or interdependence within their VA essay.

Thus, the *Independent VA* condition restricted the number of values that participants could choose to 5 values, mostly independent (*independence, learning and gaining knowledge, curiosity, government and politics*, and *being good at art*), and asked students to select 2–3 of their most important values and write why they are important. The *Interdependent VA* condition also restricted the number of values that participants could choose to 5 values, mostly interdependent (*relationships with friends and family, belonging to a social group*, and *spiritual or religious values*, *government and politics* and *being good at art*), and asked students to select 2–3 of their most important values and write why they are important. These methods are identical to those employed in previous VA research that successfully induced independent and interdependent reflection on values (Tibbetts et al., 2016a).

The *Combined VA* condition was designed to elicit both independent and interdependent writing by encouraging students to reflect on both their independent and interdependent values. In the *Combined VA* condition participants saw two columns; one column contained the values most correlated with independent writing along with one rarely selected neutral value (*independence, learning and gaining knowledge, curiosity, being good at art*) and the other column contained the values most correlated with interdependent writing along with another rarely selected neutral value (*relationships with friends and family, spiritual or religious values, belonging to a social group, government or politics*). Participants were given the same writing instructions as in the *Independent VA* and *Interdependent VA* conditions except they were instructed to select at least one value from each column (thus increasing the likelihood of circling at least one independent and one interdependent value to write about). The two columns were counterbalanced (i.e., half the participants in the *Combined VA* condition saw the independent values in the first column and the other half saw the interdependent values in the first column). No significant main effects or interactions with generational or URM status emerged on outcome measures as a result of which kind of values participants saw in the first column, *p* > 0.32.

At time 2 the two neutral values (*being good at art* and *government or politics*) were replaced with *school spirit* and *online social networking and/or gaming*, respectively, in each of the three VA conditions. In the control condition *school spirit* and *online social networking and/or gaming* were added to the existing 12 values used at time 1. The amendments to the values used at time 1 were implemented to make the assignment seem slightly different. This is consistent with methods used in previous VA interventions (e.g., Harackiewicz et al., 2014).

### Measures – Background and Demographic

We collected descriptive measures related to students’ academic preparation and employment record. Specifically, from administrative data we obtained students’ average ACT score, age, and the free/reduced lunch rate of the high schools students attended (this served as a proxy for poverty at both the school and neighborhood level). Self-report measures included whether or not students were employed during the semester, how many hours a week students worked during the semester, their annual household income (1 – 8 scale; 1 =< $15,000, 2 = $15,001–$25,000, 3 = $25,001–$35,000, 4 = $35,001–$50,000, 5 = $50,001–$75,000, 6 = $85,001–$100,000, 7 = $100,001–$150,000, 8 => $150,000), and how many advanced placement or international baccalaureate classes they took in high school.

Using the same orthogonal *Demographic* contrast model reported in Study 1A we examined differences on these background and demographic variables with a *Generational Status* contrast (CG students = +2, FG-Majority students = -1, FG-Minority students = -1) and a *URM Status* contrast (CG students = 0, FG-Majority students = -1, FG-Minority students = +1).

**Table 4** includes means for all baseline background, demographic, and psychological measures. The degrees of freedom for models testing effects on self-report income and percent of students who received financial assistance for school lunches are fewer because we did not impute for missing self-report income measures and because some high schools did not have accessible free/reduced lunch data (e.g., international schools, home-schooling).

The analysis of ACT scores revealed that FG students (*M* = 20.04, *SD* = 3.41) scored lower than CG students (*M* = 21.56, *SD* = 3.79) on the ACT, *t*(435) = 5.41, *p* < 0.001, β = -0.29, and that Minority-FG (*M* = 18.48, *SD* = 3.57) students performed significantly more poorly than Majority-FG students (*M* = 20.34, *SD* = 3.30), *t*(435) = 3.07, *p* = 0.002, β = 0.17.

A main effect of the *Generational Status* contrast on age indicating that on average, FG students (*M* = 20.23, *SD* = 3.92) were older than CG students (*M* = 19.63, *SD* = 3.30), *t*(435) = 2.15, *p* = 0.032, β = 0.12.

Both the *Generational Status* and *URM Status* contrasts were significant predictors of percent free or reduced lunch at students’ high schools. FG students (*M* = 31%, *SD* = 13%) attended more impoverished high schools compared to CG students (*M* = 28%, *SD* = 13%), *t*(321) = 3.36, *p* = 0.001, β = 0.23, and Minority-FG students (*M* = 36%, *SD* = 14%) attended significantly more impoverished high schools than Majority-FG students (*M* = 31%, *SD* = 13%), *t*(321) = 2.03, *p* = 0.043, β = -0.14.

The *Generational Status* contrast was also a significant predictor of household income, *t*(407) = 5.51, *p* < 0.001, β = -0.31, such that FG students reported a lower household income than CG students. An analysis of AP and IB classes taken also revealed a main effect of the *Generational Status* contrast indicating that FG students (*M* = 0.65, *SD* = 1.12), on average, took fewer AP and IB classes than CG students (*M* = 1.00, *SD* = 1.63), *t*(435) = 2.89, *p* = 0.004, β = -0.16. Logistic regression assessing employment status revealed that CG students (86% reported being employed) were more likely to be employed than FG students (74%), Wald = 7.51, *p* = 0.006, *B* = 0.24, but no significant effects on hours worked per week emerged as students reported working, on average, 17 h per week (see **Table 5** for correlations and descriptive statistics of background and demographic measures).

**Table 5:** Study 1B: Correlations and descriptive statistics of baseline demographic and descriptive variables.

	1	2	3	4	5	6	7	8
(1) Generational Status	-							
(2) ACT	-0.21**	-						
	*438*							
(3) Employment Status	-0.14**	0.16**	-					
	*431*	*431*						
(4) Hours worked per week	-0.06	0.08	0.72**	-				
	*415*	*415*	*415*					
(5) Percent free/reduced lunch	0.15**	-0.08	-0.14*	-0.13*	-			
	*324*	*324*	*320*	*308*				
(6) Household income	-0.27**	0.13**	0.07	-0.03	-0.14*	-		
	*410*	*410*	*410*	*394*	*304*			
(7) AP/IB classes taken	-0.13**	0.30**	0.11*	0.03	-0.08	0.10*	-	
	*438*	*438*	*431*	*415*	*324*	*410*		
(8) Age	0.08	-0.01	-0.11*	0.05	-0.06	-0.09	-0.07	-
	*438*	*438*	*431*	*415*	*324*	*410*	*438*	

*Mean*	0.15	20.69	0.58	17.19	29.71	4.47	0.80	19.97
*SD*	0.99	3.65	0.81	12.64	12.96	2.02	1.37	3.67


*Generational status and employment status were coded such that continuing-generation students = -1 and first-generation students = 1; similarly, not employed = -1, employed = 1. Italicized numbers represent *N*. ^∗^*p* < 0.05, ^∗∗^*p* < 0.01.*

Taken together these analyses indicate that FG students at the 2-year colleges came from more impoverished backgrounds and had weaker academic preparation (indexed by their lower ACT scores and fewer AP and IB classes taken) compared to CG students. Furthermore, consistent with prior intersectional research (Harackiewicz et al., 2016), Minority-FG students reported the most disadvantage in that they attended the most impoverished high schools and obtained the lowest ACT scores – even when compared to other FG (Majority) students.

### Measures – Psychological

In addition to assessing student motives, academic and social concerns, perceived match, and college belonging at the start of the semester (as reported in Study 1A) we also collected three measures specific to the 2-year college context (2-year college belonging, 2-year college identity, and 2-year college relative preparedness) at the end of the semester (approximately week 13). We measured 2-year college specific measures in order to further elucidate the FG student experience at these schools. Examining differences in students’ responses to the general college measures compared to the 2-year college specific measures could provide insight into whether or not students respond differently to questions about college in general compared to the specific 2-year college they attend. This may be particularly important in the 2-year college context, where many students are thinking about transferring to a 4-year college.

Additionally, academic and social concerns, perceived match, and college belonging were again collected at the end of the semester to measure change in these measures over time (see Study 1A for full measures).

#### Two-Year College^3^ Belonging

Two-year college belonging was measured with two items (“I belong at this 2-year college” and “I feel like this 2-year college is a good fit for me,” α = 0.92).

#### Two-Year College Identity

Two-year college identity was measured with two items (“Being a student at this 2-year college is an important part of my identity” and “I am proud to be a student at this 2-year college,” α = 0.70).

#### Two-Year College Relative Preparedness

Two-year college relative preparedness was measured with two items (“I feel more academically prepared than other students at this 2-year college” and “I sometimes feel like other students on campus have academic skills that I don’t,” reverse coded, α = 0.77).

### Results – Experimental Findings

#### Manipulation Check

In order to test whether the VA conditions induced participants to write more about independence and interdependence, relative to the *Control* condition, each essay was coded using the same holistic coding system used to code for independence and interdependent in VA essays from prior research (Tibbetts et al., 2016a). That is, each essay was read by at least two trained coders who evaluated whether each essay contained themes of independence and interdependence. Each essay was given two scores: a score of 0 or 1 for independence and a score for 0 or 1 for interdependence (0 = no, 1 = yes). Coders maintained high inter-rater reliability for both independence (Cohen’s kappa = 0.92) and interdependence (Cohen’s kappa = 0.89) coding. Initial agreement among coders was over 90% for both independence and interdependence coding and for the instances in which coders disagreed, a third coder was consulted to resolve any ambiguity. Independence and interdependence coding scores were summed across the two essays to form a single measure, ranging from 0 to 2, representing the extent to which participants wrote about independence and interdependence across both VA administrations.

#### Analysis Model

In order to examine treatment differences on independent and interdependent themes, as a function of both experimental condition and generational status, we tested a regression model that included three dummy codes using the control condition as the reference group, as well as the *Demographic* contrasts described above (*Generational Status, Minority Status*), with the addition of six interaction terms (between treatment and demographic contrasts). This 11 term model included 5 main effects (3 dummy codes, 2 *Demographic* contrasts) and 6 two-way interactions (between the 3 dummy codes and the 2 *Demographic* contrasts) to form our *basic model*.

#### Independence Coding

**FIGURE 1:**
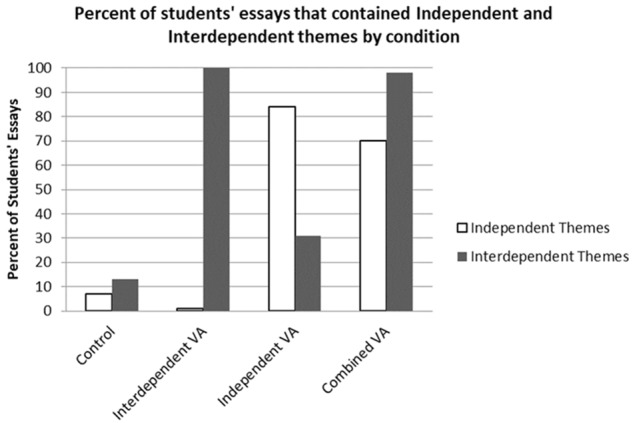
Study 1B: Percentage of essays that contained themes of independence and interdependence, as a function of values-affirmation condition.

A significant main effect of the *Independent VA* condition, relative to the *Control* condition, emerged indicating that students in the *Independent VA* condition wrote more about independence (84% wrote about independence) than students in the *Control* condition (7%), *t*(426) = 10.29, *p* < 0.001, β = 0.58. Similarly, a main effect of the *Combined VA* condition, relative to the *Control* condition emerged indicating that students in the *Combined VA* condition (70%) wrote more about independence that students in the Control condition (7%), *t*(426) = 8.44, *p* < 0.001, β = 0.53. No other significant main effects or interactions emerged. These results suggest that the Independent and Combined VA prompts were effective in encouraging students to write about independence, see **Figure 1**.

#### Interdependence Coding

A main effect of the *Interdependent VA* condition relative to the *Control* condition emerged indicating that students in the *Interdependent VA* condition wrote more about interdependence (100% wrote about interdependence) than students in the *Control* condition (13%), *t*(426) = 18.24, *p* < 0.001, β = 0.77. Similarly, a main effect of the *Combined VA* condition relative to the *Control* condition emerged indicating that students in the *Combined VA* condition wrote more about interdependence (98%) that students in the *Control* condition, *t*(426) = 18.41, *p* < 0.001, β = 0.86. Finally, a main effect of the *Independent VA* condition relative to the *Control* condition emerged indicating that students in the *Independent VA* condition wrote more about interdependence (31%) than students in the *Control* condition, *t*(426) = 2.52, *p* = 0.01, β = 0.11. No other significant main effects or interactions emerged.

The manipulation checks indicate that students did in fact write more about independence and interdependence in the *Independent VA* and *Interdependent VA* conditions, respectively. Furthermore, students wrote more about both independence and interdependence in the *Combined VA* condition and surprisingly, this was also the case in the *Independent VA* condition. Although the *Combined VA* condition successfully induced both independent and interdependent writing (relative to the *Control* condition) it is notable that 98% of students wrote about interdependence whereas only 70% wrote about independence. Additionally, given that nearly a third of students in the *Independent VA* condition also wrote about interdependence (31%), despite not being offered interdependent values to choose from, it seems that students have a natural tendency to write about interdependence that does not exist for writing about independence. It may be more difficult to encourage students to reflect on their independence (see **Table 6** for the percentage of students who selected each value by VA condition).

**Table 6:** Study 1B: Percent of students who selected each value by values affirmation condition.

Value	Independent VA	Interdependent VA	Combined VA
Independence	80%	–	60%
Learning and gaining knowledge	96%	–	80%
Curiosity	69%	–	32%
Relationships with friends and family	–	98%	93%
Belonging to a social group	–	68%	7%
Spiritual and religious values	–	44%	20%
Government and politics	5%	14%	5%
Being good at art	15%	19%	2%
School spirit	3%	1%	1%
Social networking and/or gaming	11%	16%	8%


**Because participants were instructed to choose either 2 or 3 values, percentaes do not sum to 100%. VA, values affirmation.**

#### Course Grade

Although students were randomly assigned to condition at the student level, we used hierarchical linear modeling (HLM) to account for the nested structure of the data (Raudenbush and Bryk, 2002). We tested a two-level random-intercept model in which students were nested within eleven different instructors. The intraclass correlation coefficient was small; between-instructor variance accounted for 10% of variance in course grade. Although this analysis demonstrated that the nesting of students would not have a large effect on the analyses compared to multiple regression models, we modeled the nesting structure so that accurate standard errors would be obtained. Comparison of regression and HLM results for the primary analysis on course grade are presented in **Table 7**. Analyses with HLM and regression yielded consistent results. Regression results are reported here so that effect sized (betas) can be reported.

**Table 7:** Study 1B: Regression analysis of course grade.

	Course grade					
	
	Regression			HLM		
		
Predictor	β	*t*(*414*)	*p*	γ	*F (df)*	*p*
FG students vs. CG students	-0.24	2.17	0.030	-0.17	4.92 (1, 405)	0.027
Majority-FG vs. Minority-FG	0.30	2.62	0.009	0.45	6.03 (1, 405)	0.015
Independent VA vs. Control	0.12	1.77	0.077	0.19	1.09 (1, 405)	0.298
Interdependent VA vs. Control	0.06	0.81	0.417	0.08	0.21 (1, 405)	0.646
Combined VA vs. Control	0.10	1.29	0.198	0.13	0.53 (1, 405)	0.466
FG vs. CG × Independent VA vs. Control	0.05	0.72	0.471	0.07	0.36 (1, 405)	0.547
Maj-FG. Vs. Min-FG. × Independent VA vs. Control	-0.18	2.08	0.038	-0.49	3.81 (1, 405)	0.052
FG vs. CG × Interdependent VA vs. Control	0.10	1.29	0.199	0.13	1.54 (1, 405)	0.215
Maj-FG vs. Min-FG × Interdependent VA vs. Control	-0.15	1.83	0.068	-0.45	3.34 (1, 405)	0.068
FG vs. CG × Combined VA vs. Control	0.17	1.97	0.049	0.20	3.80 (1, 405)	0.052
Maj-FG vs. Min-FG × Combined VA vs. Control	-0.14	1.45	0.148	-0.34	1.94 (1, 405)	0.164
Gender	-0.12	1.28	0.202	-0.08	0.53 (1, 405)	0.468
Gender × Independent VA vs. Control	-0.01	0.16	0.874	-0.08	0.27 (1, 405)	0.607
Gender × Interdependent VA vs. Control	-0.02	0.36	0.723	-0.12	0.62 (1, 405)	0.431
Gender × Combined VA vs. Control	0.09	1.29	0.200	0.10	0.61 (1, 405)	0.434
Course	0.13	1.42	0.155	0.15	1.00 (1, 9)	0.343
Course × Independent VA vs. Control	0.02	0.28	0.783	0.05	0.11 (1, 405)	0.736
Course × Interdependent VA vs. Control	0.07	1.07	0.288	0.17	1.58 (1, 405)	0.209
Course × Combined VA vs. Control	-0.02	0.23	0.821	-0.00	0.00 (1, 405)	0.987
ACT	0.34	3.99	0.000	0.40	20.61 (1, 405)	0.000
ACT × Independent VA vs. Control	0.04	0.70	0.488	0.08	0.32 (1, 405)	0.573
ACT × Interdependent VA vs. Control	0.00	0.06	0.947	-0.02	0.01 (1, 405)	0.900
ACT × Combined VA vs. Control	0.09	1.38	0.167	0.15	1.42 (1, 405)	0.234


**FG students vs. CG students (Majority-FG students, +1, Minority-FG students, +1, CG students, -2), Majority-FG vs. Minority-FG (Majority-FG students, +1, Minority-FG students, -1, CG students, 0), Independent VA vs. Control (Independent VA, +1, Interdependent VA, 0, Combined VA, 0, Control, 0), Interdependent VA vs. Control (Independent VA, 0, Interdependent VA, +1, Combined VA, 0, Control, 0), Combined VA vs. Control (Independent VA, 0, Interdependent VA, 0, Combined VA, +1, Control, 0), Gender (Male = +1, Female = -1), Course (biology = 1, psychology = -1)*.*

In order to examine intervention effects on course grade we used the same *basic model* that was used for the manipulation checks with the addition of three covariates: gender (female = -1, male = +1), course (psychology = -1, biology = +1; in the HLM model course was entered as a level-two predictor), and ACT. All possible interactions between the *Demographic* contrasts, the dummy codes, and gender were tested. None of the gender × *Demographic* contrast interactions terms were significant, and were therefore trimmed from the model. Interactions between the covariates and our treatment contrasts (i.e., the three dummy codes) were retained to decrease potential bias when interpreting the interactive effects of the independent variables (Yzerbyt et al., 2004). Thus the final model included 23 terms: 5 main effects (3 dummy codes, 2 *Demographic* contrasts), 6 two-way interactions (between the 3 dummy codes and the 2 *Demographic* contrasts), 3 covariates (gender, course, ACT) as well as 9 two-way interactions between the 3 covariates and the 3 dummy codes (see **Table 7** for full model).

Three significant main effects on course grade emerged. The *Generational Status* contrast revealed that CG students (*M* = 2.59, *SD* = 1.14) received higher grades than FG students (*M* = 2.39, *SD* = 1.07), *t*(414) = 2.17, *p* = 0.03, β = -0.24. The *URM Status* contrast revealed that Majority-FG students (*M* = 2.48, *SD* = 1.07) received higher grades than Minority-FG students (*M* = 1.97, *SD* = 1.00), *t*(414) = 2.62, *p* = 0.01, β = 0.30. A main effect of ACT indicated that students with higher ACT scores received higher grades in the class, *t*(414) = 3.99, *p* < 0.001, β = 0.34.

**FIGURE 2:**
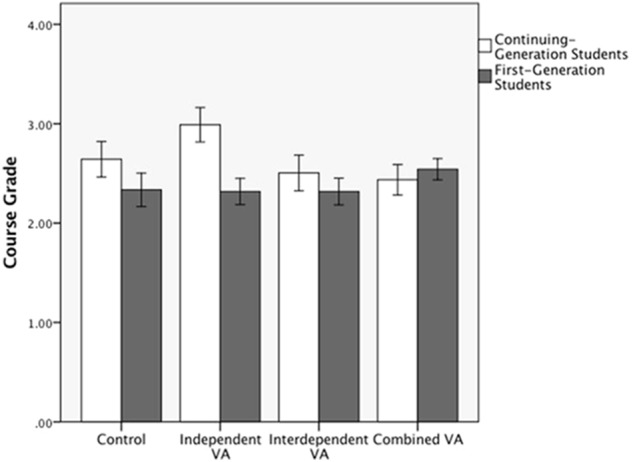
Study 1B: Mean course grade with ±1 standard error for performance among continuing-generation and first-generation students as a function of values-affirmation condition.

Two significant two-way interactions also emerged. The interaction of the *Combined VA* dummy code and *Generational Status* contrast indicated that whereas FG students performed better in the *Combined VA* condition (*M* = 2.52, *SD* = 1.00) compared to the *Control* condition (*M* = 2.33, *SD* = 1.24), CG students performed more poorly in the *Combined VA* condition (*M* = 2.47, *SD* = 1.21) compared to the *Control* condition (*M* = 2.63, *SD* = 1.14), *t*(414) = 1.97, *p* = 0.05, β = 0.17 (see **Figure 2**).

**FIGURE 3:**
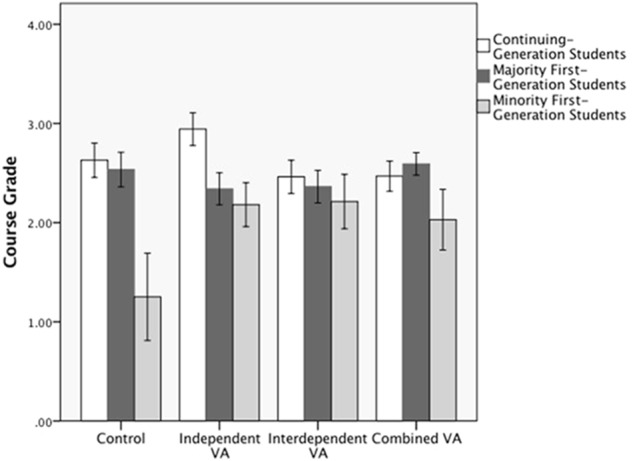
Study 1B: Mean course grade with ±1 standard error for performance among continuing-generation, majority first-generation, and minority first-generation students as a function of values-affirmation condition.

The interaction of the *Independent VA* dummy code with the *URM Status* contrast indicate that whereas Minority-FG students performed better in the *Independent VA* condition (*M* = 2.18, *SD* = 0.74) compared to the *Control* condition (*M* = 1.25, *SD* = 1.24), Majority-FG students performed more poorly in the *Independent VA* condition (*M* = 2.34, *SD* = 1.10) compared to the *Control* condition (*M* = 2.54, *SD* = 1.15), *t*(414) = 2.08, *p* = 0.04, β = -0.18 (see **Figure 3**). No other effects were significant.

#### Psychological Outcomes

**Table 8** shows the correlations and descriptive statistics of psychological outcome variables, course grade, and ACT score. The same model used to assess effects on course grade was used to examine effects on the psychological measures collected the 13th week of the class (academic and social concerns, perceived match, college belonging, 2-year college belonging, 2-year college identity, and 2-year college relative preparedness). When possible, we also controlled for baseline measures of the same construct (academic and social concerns, perceived match, and college belonging) allowing us to examine change in these measures over the course of the semester.

**Table 8:** Study 1B: Correlations and descriptive statistics of course grade, ACT, and psychological variables.

	1	2	3	4	5	6	7	8	9	10	11	12	13
(1) Course grade	-												
(2) ACT	0.41**	-											
(3) Independent motives	-0.06	-0.11*	-										
(4) Interdependent motives	-0.14**	-0.29**	0.55**	-									
(5) Academic and social concerns (baseline)	-0.08	0.02	-0.11*	-0.06	-								
(6) Academic and social concerns (final)	-0.10*	-0.00	-0.05	-0.03	0.58*	-							
(7) College belonging (baseline)	0.04	-0.08	0.39**	0.31**	-0.22**	-0.11*	-						
(8) College belonging (final)	0.24**	0.07	0.27**	0.25**	-0.13*	-0.21**	0.57**	-					
(9) Perceived match (baseline)	-0.01	-0.10*	0.37**	0.38**	-0.04	0.02	0.46**	0.27**	-				
(10) Perceived match (final)	0.18**	-0.04	0.23**	0.27**	-0.04	-0.09*	0.32**	0.45**	0.46**	-			
(11) Two-year college Belonging	0.15**	-0.04	0.13**	0.21**	-0.04	-0.10*	0.22**	0.45**	0.25**	0.64**	-		
(12) Two-year college Identity	-0.02	-0.11*	0.28**	0.35**	0.04	0.01	0.27**	0.37**	0.34**	0.56**	0.66**	-	
(13) Two-year college Relative Preparedness	0.31**	0.24**	0.02	-0.01	-0.27	-0.35	0.17**	0.28**	0.10*	0.07	0.03	-0.09	-

*Mean*	2.48	20.69	5.88	5.47	3.57	3.68	5.98	5.73	5.09	5.17	5.10	3.94	4.41
*SD*	1.10	3.65	0.97	1.12	1.37	1.26	1.13	1.25	1.18	1.29	1.40	1.28	1.43


*^∗^*p* < 0.05, ^∗∗^*p* < 0.01.*

##### Academic and social concerns

Apart from baseline academic and social concerns significantly predicting final academic and concerns, *t*(413) = 14.05, *p* < 0.001, β = 0.58, no other effects emerged.

##### Perceived match

**FIGURE 4:**
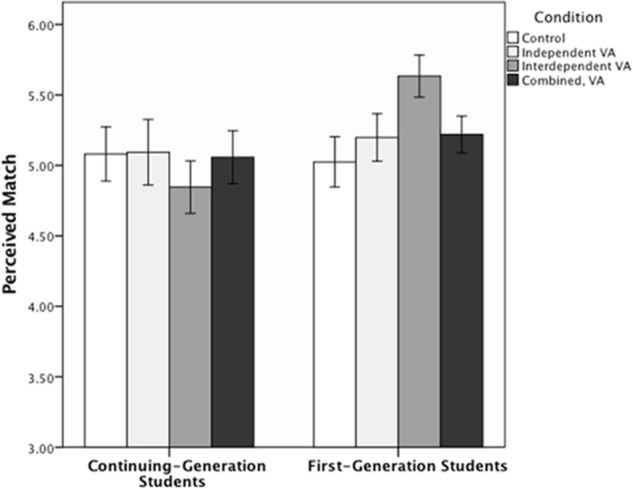
Study 1B: Mean perceived match and ±1 standard error for continuing-generation and first-generation students as a function of condition.

In addition to baseline perceived match predicting final perceived match, *t*(413) = 10.13, *p* < 0.001, β = 0.45, an interaction between the *Interdependent VA* dummy code term and the *Generational Status* contrast indicated that FG students perceived more match in the *Interdependent VA* condition (*M* = 5.69, *SD* = 1.08) compared to the *Control* condition (*M* = 5.05, *SD* = 1.32), *t*(413) = 2.13, *p* = 0.034, β = 0.16; CG students did not show this pattern (*M* = 4.86, *SD* = 1.25 in the *Interdependent VA* condition; *M* = 5.05, *SD* = 1.23 in the *Control* Condition; see **Figure 4**).

##### College belonging

In addition to baseline college belonging significantly predicting final college belonging, *t*(413) = 14.13, *p* < 0.001, β = 0.57, a main effect of ACT emerged indicating that students with higher ACT scores reported more college belonging, *t*(413) = 2.07, *p* = 0.039, β = 0.16.

##### Two-year college belonging

A main effect of ACT emerged indicating that students with higher ACT scores reported more two-year college belonging, *t*(414) = 1.93, *p* = 0.054, β = 0.18. Additionally, a significant interaction between ACT and the *Interdependent VA* dummy code term indicated that students with lower ACT scores reported more 2-year college belonging in the *Interdependent VA* condition (*M* = 5.55 for students 1 SD below the mean on ACT) than the *Control* condition (*M* = 4.93 for students 1 SD below the mean on ACT), *t*(414) = 2.18, *p* = 0.03, β = -0.14.

##### Two-year college identity

A significant main effect of the *Generational Status* contrast indicated that FG students (*M* = 4.65, *SD* = 1.36) reported higher levels of 2-year college identity than CG students (*M* = 4.08, *SD* = 1.47), *t*(414) = 2.28, *p* = 0.023, β = 0.28.

##### Two-year college relative preparedness

A significant main effect of the *Generational Status* contrast indicated that FG students (*M* = 3.76, *SD* = 1.24) reported less 2-year college relative preparedness than CG students (*M* = 4.20, *SD* = 1.30), *t*(414) = 2.01, *p* = 0.045, β = -0.24.

### Discussion of Study 1B

The results of Study 1B raise interesting questions about which values are most effective for FG students at 2-year colleges to write about in a VA intervention. Writing about interdependence and independence had different effects on our psychological measures and academic performance. For example, over the course of the semester, FG students perceived more match with their college when they completed an *Interdependent VA* assignment. FG students may have perceived more match because the writing task they were assigned emphasized the same kinds of values they strongly endorse. Receiving a writing assignment, ostensibly created by course instructors, that encourages active reflection on one’s interdependent values may implicitly signal to students that their instructors and university also value interdependence. This perceived match with university may be, in part, why FG students did not report the same belonging concerns typically reported by FG students at 4-year institutions. In fact, FG students reported higher levels of 2-year college identity suggesting that the culture of 2-year colleges may be easier for FG students to relate to compared to their CG student peers. However, it is also interesting to note that FG students performed better in their courses when they had the opportunity to write about independent values.

Indeed, the strongest VA effects in the present study indicate that FG students performed better in the *Combined VA* condition than the *Control* condition, relative to CG students, and that Minority-FG students performed better in the *Independent VA* condition than the *Control* condition relative to Majority-FG students. One commonality between the *Combined VA* and the *Independent VA* conditions was the opportunity to affirm independent values (e.g., *learning and gaining knowledge, curiosity, independence*). Previous research at 4-year institutions would suggest that affirming these independent values may be important to overcoming a cultural mismatch (Tibbetts et al., 2016a). However, in the present study, no evidence of cultural mismatch exists and yet we still observed treatment effects on grades for FG students in conditions that promoted independent values. It may be that in addition to, or instead of, overcoming a cultural mismatch, affirming independence has the ability to help college students more generally.

## General Discussion

One goal of the present research was to examine the nature of cultural mismatch at 2-year colleges. The results suggest that FG students in the current sample may not experience the identity threats and belonging concerns associated with cultural mismatch, that FG students at 4-year universities contend with. According to cultural mismatch theory, FG students experience these threats when their motivations and the norms and values implicit in the university culture differ. Whereas previous research on cultural mismatch has noted that the interdependent motivations of FG students are inconsistent with the independent values championed by selective colleges and universities, the present study indicates that the norms of the 2-year colleges may be better matched for FG students. Indeed, administrators at the 2-year colleges were more likely to characterize their schools as possessing more interdependent norms and values than independent. The interdependent characterization of institutional norms would be consistent with the interdependent motivations that FG students have been shown to possess both here and in previous research (e.g., Stephens et al., 2012a; Harackiewicz et al., 2014; Tibbetts et al., 2016a). Furthermore, the fact that FG students perceived more match with their college when they completed an *Interdependent VA* assignment suggests that students may also perceive the culture of 2-year colleges to be more interdependent than independent. Consistent with prior research (e.g., Stephens et al., 2012b), an interdependent perception of the college context may be experienced as more comfortable and less stressful for FG students and may be why FG students in the current sample did not report significantly less belonging than CG students.

Even though FG students may perceive a more inclusive and welcoming culture at the 2-year colleges compared to a typical 4-year institution, a social-class achievement gap still persists. Whereas previous research posits that the social-class achievement gap may be driven, in part, by a cultural mismatch (Stephens et al., 2012a), this logic does not seem to apply to the present context, in which FG students and instructors similarly endorsed interdependent norms and values. Given that we failed to document a cultural mismatch, but did find evidence of the social class achievement gap, the question remains: what is the cause of the social-class achievement gap in this context, and what are the implications for intervention?

Some administrative and self-report data we collected may provide some insight. In addition to being less academically prepared (e.g., lower ACT scores, fewer AP/IB credits taken in high school), FG students reported fewer economic resources. They attended high schools with higher poverty rates (indexed by percent of students receiving free and/or reduced lunch rates) and reported less household income compared to CG students. Furthermore, Minority-FG students reported the most disadvantage in that they attended the most impoverished high schools and came to the 2-year colleges with, on average, the lowest ACT scores. Considering the relative disadvantage of these students, along with the fact that 79% of the present sample was also employed during the school year (compared to the national average employment rate of 40% for full-time college students; Snyder and Dillow, 2015), it becomes apparent these students face a number of barriers to their academic success.

### The Connection Between Independence and Academic Values

If values affirmation did not help FG students’ academic performance by addressing a cultural mismatch is it possible that it worked by alleviating the effects of some other barrier to FG students’ success? The fact that FG students performed better when given the opportunity to write about independent values may provide some tentative insights. The majority of students in the present sample live at home with parents who never attended college. Whereas students at 4-year colleges typically move away from home and are thus forced to be more independent when they attend college, most students at the 2-year colleges do not have that experience. Given that college students can likely benefit from some amount of independence, it may be that having 2-year college students reflect on their independent values promotes the kinds of independent behaviors that 4-year students have already found to be adaptive for college success (e.g., independent study time).

Furthermore, the three independent values that students could select in the treatment conditions are all associated with academic traits that could be beneficial to those striving to obtain a college degree. For example, reflecting on the importance of *learning and gaining knowledge*, may prove particularly motivating for students who do not have parents that can readily model the behaviors typically required for college learning. For students who may be unaccustomed to thinking about the value of education, writing an essay about the importance of *curiosity, learning and gaining knowledge*, and *independence* could prove to be a powerful motivator.

This implicit connection between independent and academic values may account for the improved performance of students when they select independent values to affirm. Indeed, this is consistent with positive main effects of independent writing on academic performance noted in previous research (Tibbetts et al., 2016a). Whereas prior VA research has argued against affirming academic values in academic settings in fear that it might exacerbate threat concerns for disadvantaged students (e.g., Sherman and Cohen, 2006), more recent research suggests that this line of thinking could be less relevant in college (e.g., Kizilcec et al., 2017a,b). For example, the original tenets of affirmation theory posited that observed VA effects were induced by participants affirming core aspects of their identity that were unrelated to the threatening domain they were being evaluating in (e.g., Sherman and Cohen, 2006). Affirming parts of one’s identity outside of a potentially threatening domain (e.g., affirming your sense of humor in school) was thought to reestablish alternative self-resources important for maintaining self-integrity in times of stress. In fact, previous research explicitly argued against affirming values that were relevant to the threatening domain. Affirming values or aspects of the self that are related to the domain of threat was thought to increase the salience of the threat thereby exacerbating its negative effects.

Although the independent values driving VA effects for FG students may be implicitly academic and therefore related to the domain of potential threat (e.g., college), we see no evidence that affirming independence negatively affects FG student outcomes. Furthermore, recent VA research corroborates the notion that affirming within the domain of threat may be beneficial in college courses (Kizilcec et al., 2017a,b). Disadvantaged students enrolled in a massive open online course (MOOC) benefited from an affirmation intervention that asked them to write about how enrolling in the course reflected and reinforced their most important values. Contrary to previous research, these studies explicitly tied students’ important values to the domain of potential threat (their classes) but still demonstrated positive effects on grades for threatened students.

### Belonging at 2-Year Colleges

An additional finding from the present work relates to the meaning of belonging at 2-year colleges and how it raises issues not typically observed at 4-year institutions. Students generally believe they belong in college if they possess the required skill to succeed. For example, belonging measures are often positively correlated with how well students believe they can perform (e.g., Harackiewicz et al., 2014). At the 2-year colleges, however, this may not always be the case. Some students (e.g., CG students) may report low levels of belonging at 2-year colleges, not because they lack the skills necessary to succeed, but because they view a 4-year college as the ultimate goal and are thus less concerned with belonging at the 2-year school. In other words, these students likely perceive 2-year colleges as a stop-over between high school and the 4-year college they eventually want to attend. This may be particularly true of CG students whose parents have already paved a path to a baccalaureate degree that their children are expected to follow and could explain why CG students reported lower levels of 2-year college identity than FG students but significantly higher levels of 2-year college relative preparedness. If CG students feel that they are more academically prepared than the students around them, they may be less likely to identify with 2-year colleges or report a sense of belonging at these schools. Given the preponderance of interventions focused on student belonging, it is important that researchers understand what kind of belonging is important for the contexts they are working in. For example, is 2-year college belonging or general college belonging more critical for the academic success of 2-year college students? It may be that belonging at the 2-year colleges is important for retaining students at the 2-year colleges but that general college belonging is more critical for academic outcomes at 4-year institutions.

### Future Directions

Given that different kinds of belonging may be differentially related to academic outcomes, future research should explore which forms of belonging impact the various aspects of the college student experience.

A second future direction that cultural mismatch research should explore is how student motives may vary by type of academic institution. For example, whereas CG students have been shown to more strongly endorse independent motivations and values compared to FG students, we find a different pattern in that FG students more strongly endorse independent motivations for attending college compared to CG students. To our knowledge, this is the first study to document a stronger endorsement of independent motives for FG students compared to CG students, suggesting that the motivational characteristics of different student groups varies by academic institution. This also suggests that the field may benefit from further explicating how the interaction between student and university values affect student belonging. For example, if CG student motives are shown to be discrepant from university norms, do they experience a cultural mismatch themselves or does their educational background protect them from the belonging concerns that typically arise from an experienced mismatch? It is also worth noting that in the current sample, Minority-FG students more strongly endorsed both independent and interdependent motives even when compared to other Majority-FG students, highlighting the importance of considering students’ multiple identities when implementing interventions. If a goal is to implement interventions that target specific psychological processes (Walton, 2014; Tibbetts et al., 2016b; Harackiewicz and Priniski, 2018), it is imperative that we understand how different psychological and motivational patterns vary as a function of the students’ multiple identities.

A third future direction relates to how we can most effectively structure reflective writing activities (like VA) to give students the best chance of benefitting from their writing. If future research continues to demonstrate the effectiveness of reflecting on academic values (e.g., *learning and gaining knowledge*) then perhaps we can structure writing prompts that directly prompt this kind of writing (i.e., Describe a time you achieved a personal goal on your own) to maximize the effectiveness of reflective writing.

A fourth future direction concerns the nature of the control condition. Given that students write about other people’s values in the control condition, it may cause students to reflect on their interdependence. Indeed, more than 10% of control participants affirmed their interdependence (see **Figure 1**). If future researchers want to test the effectiveness of an *interdependent VA* condition against a neutral control condition, it may be necessary to alter the standard control condition traditionally used in VA research.

Finally, it is also important to point out that there were some hints that VA could harm some majority students’ academic performance. For example, although FG students performed best in the *Combined VA* condition, CG students tended to perform worse in the *Combined VA* condition than the *Control* condition (albeit, not significantly worse). Similar patterns for majority students have been found in previous VA research. For example, a previous VA intervention implemented in a college physics class found that although values affirmation improved women’s exam scores to close a gender gap in the course, men performed worse in the VA condition compared to the Control (Miyake et al., 2010). Similarly, Shnabel et al. (2013) noted that although writing about interdependence mediated VA effects on grades for African American middle schoolers, it had a negative effect on white students’ GPAs. If VA interventions are going to be implemented at scale, it is important to gain a better understanding of why it improves outcomes and for whom it is most effective. By varying the kinds of values students affirm and testing them across different academic institutions researchers should be able to accumulate enough knowledge to make recommendations about what kinds of VA interventions may be most effective for a given context.

## Limitations

There are also several limitations to consider when evaluating these results. For one, the number of instructors surveyed in Study 1A to assess institutional norms was small (*N* = 18), and unlike Stephens et al. (2012a) we did not survey administrators. However, we believe that assessing the independent/interdependent climate of 2-year schools (e.g., community colleges) is critical to fully understanding the nature and implications of cultural mismatch theory.

Another limitation is that we did not include a *Standard VA* condition (i.e., the traditional VA condition that includes a list of 12 values for students to write about in the treatment condition). A *Standard VA* condition would have allowed us to test the observed effects against a condition commonly used in previous VA studies and enabled us to evaluate if writing about both independence and interdependence is more effective for students when they are explicitly instructed to do so (as in the *Combined VA* condition) or freely choose to do so (as they might in a *Standard VA*). Power considerations precluded us from including this condition, but it will be important to include in future interventions, to allow more direct comparisons with previous research.

Similarly, the lack of diversity in the present sample precluded us from examining the data with a more intersectional approach. For example, we had a low number of Minority-CG students (*N* = 12) making full intersectional analyses impossible.

## Conclusion

The present findings highlight the potential benefits of future research on cultural mismatch theory and values affirmation interventions. If we are to examine the full nature of cultural mismatch theory and maximize the potential of future VA interventions, it is imperative that we understand more about the contexts of higher education and the students we intend to serve. By expanding the research of cultural mismatch theory and VA interventions to novel contexts we can more effectively help students with a deeper and more nuanced understanding of the critical issues they face.

## Ethics Statement

This study was carried out in accordance with the recommendations of the Education and Social/Behavioral Science IRB at the University of Wisconsin–Madison. The protocol was approved by the Institutional Review Board.

## Author Contributions

YT collected and analyzed the data in addition to writing the manuscript. SP and CH collected the data, helped to analyze the data, and edited the manuscript. GB helped to fund the project and construct the experimental design. JH consulted on the experimental design, data collection, and manuscript-writing process.

## Conflict of Interest Statement

The authors declare that the research was conducted in the absence of any commercial or financial relationships that could be construed as a potential conflict of interest.

## Footnotes

1 “Two-year colleges system X” was replaced with the actual name of the 2-year college system on the survey; we replaced it in this manuscript to protect anonymity.

2 When we examined differences between Majority CG and Minority CG groups no significant differences emerged on any of the reported variables. A trend on independent motives indicated that Minority CG students (*M* = 6.29, *SD* = 0.70) more strongly endorsed them compared to Majority-CG students (*M* = 5.79, *SD* = 0.93), *p* = 0.08, but no other effects approached significance, *p* > 0.21.

3 “Two-year college” was replaced with the actual name of the 2-year college system on the survey; we replaced it in this manuscript to protect anonymity.
